# A prospective comparison of 3 hamstring ACL fixation devices—rigidfix, bioscrew, and intrafix—randomized into 4 groups with a minimum follow-up of 5 years

**DOI:** 10.1186/s12893-022-01685-x

**Published:** 2022-06-30

**Authors:** Leena Metso, Ville Bister, Jerker Sandelin, Arsi Harilainen

**Affiliations:** 1 Health Care Center of the City Helsinki, Työpajankatu 14 A, 00580 Helsinki, Finland; 2 Helsinki University Hospital Trauma Unit, Töölö Hospital Topeliuksenkatu 5, 00260 Helsinki, Finland; 3 Department of Surgery, Clinicum, Faculty of Medicine, University of Helsinki, Helsinki, Finland; 4 ORTON Orthopaedic Hospital, Tenholantie 10, 00280 Helsinki, Finland

**Keywords:** Anterior cruciate ligament reconstruction, Clinical outcome, Hamstring tendon, Fixation method

## Abstract

**Background:**

ACL (anterior cruciate ligament) reconstruction remains the gold standard surgical option for patients with ACL tears. There are many fixation devices available for ACL reconstruction. Recent ACL reconstruction strategies are aiming to reproduce the native anatomy and normal kinematics of the knee. This is a five years follow-up report of some of the new devices for graft fixation. A two years follow-up data was published previously.

**Methods:**

120 patients were randomized into four different groups (30 each) for ACL reconstruction with hamstring tendons: group I femoral Rigidfix cross-pin and Intrafix tibial extension sheath with a tapered expansion screw; group II Rigidfix femoral and BioScrew interference screw tibial fixation; group III BioScrew femoral and Intrafix tibial fixation; group IV BioScrew fixation into both tunnels. The evaluation methods were clinical examination, knee scores, and instrumented laxity measurements.

**Results:**

In this 5 years follow-up there were 102/120 (85%) patients available, but only 77 (64,2%) attended the clinical examinations. No significant difference between the groups in the clinical results was detected. Between the 2 and 5 years follow-up there were 6 additional procedures in group I and one in group II. There was a significant difference in additional procedures between group I and the other groups (P = .041).

**Conclusion:**

There was a statistically significant difference in the additional procedures, most in group I (six). The ACL grafts were intact. Other statistically or clinically significant differences in the 5 years follow-up results were not found.

**Study design:**

Randomized controlled clinical trial; Level of evidence, 1.

*Trial registration* ISRCTN registry with study ID ISRCTN34011837. Retrospectively registered 17.4.2020.

## Background

Knee injuries are common in physically active people. Anterior cruciate ligament (ACL) injuries result in approximately 150 000 graft reconstructions in the United States every year [1]. Arthroscopic reconstruction of the ACL has become a standard procedure in orthopaedic surgery. The use of hamstring tendon grafts, semitendinosus and gracilis, (STG) as a free autograft to replace a ruptured ACL is a viable choice for a graft [2, 3].

The use of soft tissue grafts requires rigid fixation and sufficient stiffness to restore the ACL function successfully. To perform an operation with long standing good results, it is important to choose the best fixation device for that particular patient group. Surgeons can, also, use devices that they are most familiar with, without negative influence in clinical outcomes. It is important that the fixation device is structurally stable and secure until incorporation of the graft has occurred.

The purpose of this study was a prospective comparison of hamstring tendon ACL reconstructions and to report the results minimum of 5 years after surgery.

We hypothesize that all 4 techniques, described below, provide equal results in restoring knee stability and patient satisfaction with minimum follow-up of 5 years.

## Methods

The fixation method was randomized into 4 groups using Rigidfix crosspin (DePuy Mitek) in the femur and Intrafix expansion sheath and a tapered expansion screw in the tibia (DePuy Mitek). These methods were compared to interference screw technique (BioScrew; Linvatec, Largo, Florida).

**Fig. 1:**
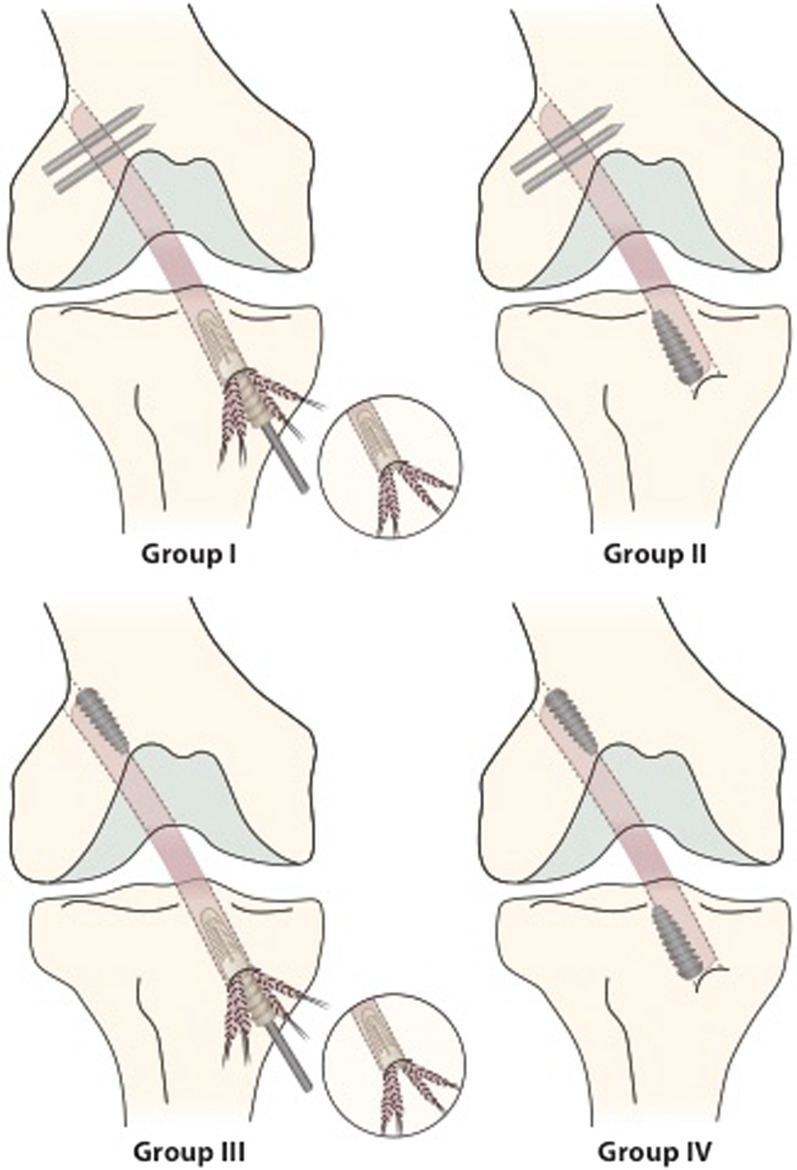
Group I: femoral Rigidfix cross-pins and a tibial expansion sheath with a tapered expansion screw Intrafix were used. In group II, femoral Rigidfix and tibial interference screw fixation with BioScrew were used. In group III, femoral BioScrew and tibial Intrafix fixation was done. In group IV, BioScrew fixation into both tunnels was employed

In group I, femoral Rigidfix cross-pins and a tibial expansion sheath with a tapered expansion screw Intrafix were used. In group II, femoral Rigidfix and tibial interference screw fixation with BioScrew were used. In group III, femoral BioScrew and tibial Intrafix fixation was done. In group IV, BioScrew fixation into both tunnels was employed. (Fig. 1).

### Patients

Patients for this study were recruited between August 2001 and August 2004 in Orton Orthopaedic Hospital Helsinki, Finland. Every patient with an ACL injury was chronologically recruited, there were no refusals. We did not collect or record the numbers nor reasons of the excluded cases.

120 patients with acute or chronic ACL injury were selected and they were randomized into 4 treatment groups, with different fixation methods [4]. Table 1 presents the demographic variables of the 4 groups. (Table 1).

**Table 1:** Demographic Variables in ACL Reconstruction in 4 Randomized Groups [4]

	Rigidfix/Intrafix	Rigidfix/BioScrew	BioScrew/Intrafix	BioScrew/BioScrew	P value
Median age, y (range)	31 (18–50)	29 (18–50)	35 (20–48)	32 (18–49)	p = 0.86, ns
Gender, F/M	11/19	11/19	12/18	10/20	p = 0.44, ns
Median interval from injury to surgery (range)	4 mo (1 wk–10 mo)	4 mo (1 wk–2 y, 8 mo)	3.5 mo (1 wk–2 y, 11 mo)	3 mo (1 wk 8 y, 3 mo)	p = 0.11, ns
Medial/lateral menisci resection	5/2	8/3	7/4	7/3	p = 0.27, ns
Medial/lateral menisci repair				/1*	p = 0.08, ns

*F* female, *M* male

*Protocol deviation

As the basis for the randomization and fixation of the graft, three devices were chosen. Femoral fixation was performed by one of the two devices, either 2 Rigidfix bioabsorbable rods (PLLA) or a BioScrew, a L-lactic acid, bioabsorbable interference screw. The length of the PLLA rod was 42 mm and the diameter was 3.3 mm. Fixation to the tibia was done with a nonabsorbable Intrafix device, which consists of an expansion sheath made from high-density polyethylene and a tapered expansion screw molded from Delrin (DePuy, Mitek), or a BioScrew. In group I, femoral Rigidfix cross-pins and a tibial expansion sheath with a tapered expansion screw (Intrafix, n = 30) were used. In group II, femoral Rigidfix and tibial interference screw fixation (BioScrew, n = 30) were used. In group III, femoral BioScrew and tibial Intrafix fixation (n = 30) was done. In group IV, BioScrew fixation into both tunnels (n = 30) was employed.

According to the study protocol, the inclusion criteria were acute or chronic (less than 5 years old) unilateral ACL tears in female or male patients, with an age range of 18 to 50 years. Additional injuries meeting the inclusion criteria were an uncomplicated meniscal lesion, small tears (flaps) which were easily dealt with and minor chondral damage, at the most grade 2 Outerbridge small lesions.

The case was excluded if the interval between the injury and surgery exceeded 5 years or ACL reconstruction was a revision procedure. Concomitant grade II to III collateral or posterior cruciate ligament tears, peripherally detached meniscal tears as well as Outerbridge 3 to 4 chondral damage and arthrosis of the knee excluded the case, also. Detached meniscal lesions needing refixation were excluded as it would be difficult to differentiate symptoms related to the refixation from those of ACL reconstruction.

Randomization was done by sealed, numbered envelopes containing information of the treatment group, and the patients as well as physiotherapists were blinded to the method used. Numbers were generated by a computer program using block randomization to ensure equal distribution of patients into each group. The diagnosis of an ACL tear was made clinically, by magnetic resonance imaging (MRI) examination when needed, and confirmed at arthrocsopy. The envelope was not opened until arthroscopy had confirmed the diagnosis after graft harvesting was completed. The two surgeons (AH, JS) were obviously not blinded to the method used.

There were altogether 4 violations to the study protocol. One randomization was misread to group III when it should have been in group II. Therefore analysis of the results was done according to the intention to treat principle keeping the patient in the intended treatment group. The data were also analyzed according to the actual fixation method with no change in the results. Against the study protocol in one case, a contralateral ACL injury was also noted 6 months after surgery (group III).

### Surgery

A diagnostic arthroscopy and minor menisci procedures were performed; 40 meniscal resections and one meniscal repair. After this, the two hamstring tendons (STG) were harvested through a vertical incision situated medial to the tibial tuberosity. Harvesting was made with a Linvatec graft harvester. The grafts were of a diameter from 7 to 10 mm. One graft diameter in the Rigidfix/Intrafix group was not recorded. There were no significant differences between the four groups regarding the graft size.

After opening the treatment allocation envelope, the grafts were individually prepared for the randomized, specific fixation methods.

The drill tunnels with femoral Rigidfix fixation were made transtibially into the posterior fourth quadrant of the femoral condyle in the sagittal plane and into 10:30-o’clock position in the right and 1:30-o’clock position in the left knee in the frontal plane. In the tibia, sagittally, the drill guide was positioned intra-articularly at the ACL footprint with the drill guide targeted in the second quadrant from anterior to posterior. No C-arm was used.

For the Rigidfix femoral fixation groups the graft was constructed, according to the manufacturer’s instructions, using whipstiches of No. 1 Vicryl (Ethicon Inc, Johnson&Johnson, Somerville, New Jersey) to join the doubled limbs of the semitendinosus and gracilis tendons together. The tunnels were made with the use of a drill guide, the depth of the transtibially drilled femoral tunnel was 30–40 mm. With the Rigidfix instrumentation, 2 transverse tunnels were drilled to receive the fixation devices. After the graft was passed into the drill tunnel by pulling it with No. 2 Vicryl loop left in the graft end, 2 Rigidfix implants were tapped through the drill guide sleeves transfixing the graft and advanced to the bone medial to the drill tunnel (Fig. 1).

For the Intrafix tibial fixation, No. 1 absorbable whipstich in the graft ends was used. The graft was spread into 4 quadrants between the sleeve and the drill tunnel. After cycling the knee 10 to 15 times, the graft was tightened. The expansion sleeve and the screw were introduced concentrically by which the 4 limbs of the graft could be compressed between the bone tunnel and the device (Fig. 1). The screws used were of three different sizes: if the graft was less than or equal to 8 mm, the 6–8 mm screw was used (7–9 mm screw if the bone quality was suboptimal), and if the graft was larger than 8 mm, the 8–10 mm screw was used.

In the BioScrew fixation, the femoral drill tunnel was performed with the “outside-in” technique using the rear-entry guide (Linvatec). A tight whipstich with No. 1 Vicryl was used to join the graft limbs together to form a single bundle. The bioabsorbable interference screw of 30 mm length and diameter equal to the graft and tunnel was inserted eccentrically, compressing the graft agaist the bony tunnel wall (Fig. 1).

If the bone was soft and the surgeon felt that the screw did not tighten sufficiently, the fixation was reinforced with a resorbable button by which the whipstich sutures were secured outside the femoral and/or tibial tunnel. If the graft ends protruded from the tibial tunnel entrance, the screw fixation was supplemented with a staple (Richards; Smith&Nephew, Memphis, Tennessee). (Table 2).

**Table 2:** Back up fixations in the four study groups

Back up fixation Group#/Name	Button Femur/Tibia	Staple Femur/Tibia
I/Rigidfix/Intrafix	0/0	1/0
II/Rigidfix/Bioscrew	1/2	0/27
III/Bioscrew/Intrafix	14/0	0/0
IV/Bioscrew/Bioscrew	9/13	2/14

Before the tibial fixation the knee was cycled 10–15 times through the range of motion in all 4 groups. The graft was then tightened to 40-N force, as instructed by the manufacturer, with the Mitek tensioning device (Intrafix fixation), and in the BioScrew fixation a manual estimate of 40-N was done. The knee was held as commonly recommended in 30° flexion, and the distal ends of the graft were secured according to the group specified.

### Post-operative care

Postoperatively no knee brace was used. Knees were mobilized immediately in the same way in all 4 groups. Crutches were in use for the first 4 weeks. Full weightbearing was allowed after 2 weeks. Active quadriceps activity, e.g. exercise cycle and exercises in water, was allowed from 3 to 4 weeks after the operation. Straigth leg raise was allowed at 6 weeks and jogging at 12 weeks after the operation. Return to sports was allowed after 6 months, contact sports and downhill skiing from 9 months postoperatively. After one year postoperatively there were no restrictions. The physiotherapist used closed kinetic chain muscle rehabilitation principles in patient guiding.

### Evaluation methods

Clinical examination was performed preoperatively and at 1, 2, and a minimum of 5 years postoperatively by the not blinded two surgeons (AH, JS). The Lachman and pivot-shift tests were graded negative I (pivot-shift: glide), slightly positive II (pivot-shift: shift), and clearly positive III with no end point (pivot-shift: clunk). Anteroposterior knee laxity (side-to-side difference with 35-pound and manual maximum force; KT-2000 arthrometer, MEDmetric Corporation, San Diego, California) and isokinetic peak muscle torques of the knee extensors and flexors at 60 deg/s and 180 deg/s (Lido MultiJoint II, West Sacramento, California) were measured, also.

The knee scores were completed by the patients: Tegner activity level was used to define the actual physical performance (1–10), for subjective evaluation Lysholm knee scores (0–100) was used. The subjective International Knee Documentation Committee (IKDC) score was calculated based on the information gathered by the 2000 IKDC KNEE EXAMINATION form. For another subjective evaluation the “patellofemoral scores” (KPS 0–100) was used, also. [5–8].

### Statistical analysis

Statistical analysis was done using the BMDP statistical package (Statistical Solutions Ltd, Cork, Ireland). The parametric data between the 4 groups were evaluated with analysis of variance. The nonparametric data were evaluated with chi-square (between the groups) and with McNemar’s or sign test (comparison over time within a group). The minimum level of significance was P = 0.05. Lysholm score was one of the main outcome measurements used. With a difference of 10 points, a clinically significant difference between the treated groups would be acknowledged. When type I error (alfa) was set at 0.05 and power (beta error, power 1-beta) at 80%, sufficient total computerized sample size would be 54 for 2 treatment groups, as calculated by the computer program. Based on our power calculations (Lysholm, 10 points difference, SD 13) the planned minimum sample size was 4 × 27 = 108 patients. 102 patients participated in the 5-year follow-up and the SD of all patients combined in Lysholm score was 12 i.e. slightly smaller than in our power calculations. Therefore, the power of our study was on same level as planned and the risk of type II error was not evident.

## Results

Patients were recruited and randomized to the study groups between August 2001 and August 2004. There were 107 patients (89.2%) available for evaluation at the 2 years follow-up. No significant clinical or statistical differences were observed between the groups.

The addition of back up fixations with a button or a staple was done if the surgeon thought it to be necessary. (Table 2) In our analysis of back up fixations on the end results (Tegner, Lysholm, IKDC, KPS), there was no correlation to better or worse result.

In the 5 years follow-up there were 102/120 (85%) patients. In group I 27, group II 22, group III 29, and in group IV 24 patients were available for analysis. 5 patients were lost from 2 to 5 years follow-up. (Table 3).

**Table 3:** Number of patients attending the 2- and 5-years follow-up examinations

	Rigidfix/Intrafix	Rigidfix/BioScrew	BioScrew/Intrafix	BioScrew/BioScrew
Number randomized	30	30	30	30
2-year follow-up	28	25	29	25
Revision before 2-year follow-up	1			2
5-year follow-up	27	22	29	24
Additional procedure before 5-year follow-up	6	1		

Preoperatively there was a higher Tegner activity level in the Rigidfix/Intrafix group [4] (Table 4).

**Table 4:** Tegner activity level, lysholm, IKDC, and patellofemoral scores (KPS) preoperatively, 2 and 5 years postoperatively

Test score (Range)	Rigidfix/Intrafix	Rigidfix/BioScrew	BioScrew/Intrafix	BioScrew/BioScrew	P-value
Tegner preop	4 (1–6)	3 (0–6)	3 (1–4)	3 (1–7)	0.024
Tegner 2 yrs	6 (3–9)	7 (3–10)	6 (3–9)	7 (3–9)	0.954
Tegner 5 yrs	6 (4–8)	6 (3–10)	6 (3–8)	6 (4–9)	0.866
Lysholm preop	75 (56–100)	77 (36–92)	75 (53–89)	78 (33–95)	0.686
Lysholm 2 yrs	94.5 (65–100)	94 (60–100)	94 (46–100)	95 (83–100)	0.628
Lysholm 5 yrs	96 (54–100)	90 (32–100)	91 (54–100)	94 (75–100)	0.524
IKDC preop	59.7 (46–83)	47.2 (31–78)	55.2 (33–76)	55.1 (29–88)	0.439
IKDC 2 yrs	91 (59–100)	91 (63–100)	91 (71–100)	90 (70–100)	0.416
IKDC 5 yrs	88 (34–100)	86 (30–100)	86 (43–100)	91 (79–100)	0.550
KPS preop	81 (47–95)	76.5 (44–93)	79 (38–91)	79 (42–98)	0.341
KPS 2 yrs	98 (82–100)	95 (95–100)	95 (95–100)	96 (96–100)	0.716
KPS 5 yrs	95 (80–100)	93 (39–100)	94 (52–100)	96 (89–100)	0.553

Otherwise there were no differences between the study groups preoperatively regarding the Lysholm or IKDC classification or patellofemoral (KPS) scores [4]. (Table 4).

### Range of motion

There was no difference between the groups in the range of motion (ROM) at the 5 years follow-up. The ROM was measured from 141° to 143° in the four groups. There was no statistically significant difference between the four groups (P = 0.699). Extension deficit was from 0° to 3° in all four groups.

### Stability

All the patients had clearly positive Lachman and pivot-shift tests preoperatively [4]. At the 5 years follow-up there were two clearly positive results on both these tests, one in the Rigidfix/Intrafix and one in the BioScrew/Intrafix group. (P = NS).

The mean preoperative manual maximum KT-2000 arthrometer side-to-side laxity difference in the four groups ranged from 4.4 mm to 5.3 mm [4]. At the 5 years clinical evaluation in the BioScrew/BioScrew group IV the manual maximum side-to-side laxity difference was the lowest at 1.6 mm (SD 3.3). In the other three groups the laxity differences were 2.1, 2.7, and 2.9 mm, respectively. There was no statistically significant difference between the groups in terms to clinical stability evaluation at the 5 years follow-up.

### Subjective evaluation

There were no differences between the groups in the 5 years follow-up examinations in Tegner activity level, Lysholm knee score, IKDC score, or the Kujala patellofemoral score (KPS). (Table 4).

The Lysholm knee score and IKDC score seemed to diminish from 2 to 5 years follow-up apart from group I. In group I the Lysholm knee score improved from 94.5 to 96. In group II it decreased from 94 to 90, in group III from 94 to 91, and in group IV from 95 to 94. At the same interval the IKDC score decreased in group I from 91 to 88, in group II from 91 to 86, in group III from 91 to 86. In group IV the IKDC score improved from 90 to 91. There were, however, no significant statistical differences found between the groups. (Table 4).

### IKDC classification

There were no statistically significant differences between the groups, nor within the groups in the 2 or 5 years follow-up examinations.

### Isokinetic peak muscle torque

Preoperatively in the BioScrew/Intrafix and the BioScrew/BioScrew groups there was a statistically significant difference compared to the other groups regarding the isokinetic peak muscle torque (percentage control-injured limb). In the above mentioned groups, there was a higher 180° /sec flexion torque observed (P = 0.032). This statistically significant difference was seen in the 2 years follow-up, also (P = 0.029) but at the 5 years follow-up there was no significant difference seen (P = 0.764).

### Complications and additional procedures

Before the 2 year follow-up, there were 3 revision ACL reconstructions [4]. Between the 2 and 5 years follow-up, there was one revision ACL reconstruction.

There were 7 additional procedures between the 2 and 5 years follow-up. Six in the RigidFix/IntraFix group I and one in the RigidFix/BioScrew group II. There was a statistically significant difference comparing group I to the other three groups (P = 0.041). In the RigidFix/IntraFix group the additional procedures between 2 to 5 years follow-up were; in two patients tears in medial meniscus were resected, one patient had the Intrafix screw removed because of protrusion and in another procedure scar tissue was removed, one patient was treated for a lateral meniscus tear with a partial resection, and one patient had an unidentified meniscus procedure performed in another hospital. In the RigidFix/BioScrew group one patient had an additional procedure to remove the Richards staple. One patient in the Bioscrew/IntraFix group had had a new injury and a revision ACL reconstruction operation. (Table 5).

**Table 5:** Additional Procedures Before 2- and 5-Years Follow-Ups

	Rigidfix/Intrafix	Rigidfix/BioScrew	BioScrew/Intrafix	BioScrew/ BioScrew
Revision	1 before 2-year follow-up		1 new injury between 2 and 5 year follow-ups	2 before 2 year follow-up
Additional procedures	6	1		

We wanted to differentiate revisions and additional procedures. Revision means a failed graft with a re-reconstruction done, additional procedures were mainly implant related surgeries and meniscal resections without an adverse effect to the result.

## Discussion

In accordance with our hypothesis, the most important finding of this study was that the results of the four fixation groups did not show any difference at the 5 years follow-up. All of these fixation devices according to this study are safe to use and provide equally good clinical results.

Graft fixation has been considered the weakest link of the reconstruction postoperatively and in the early phases of the rehabilitation [9]. Until incorporation of the graft into the bony tunnel has occurred, the strength of the graft is almost solely dependent on the way the graft is fixed into the bony tunnels [10]. Reconstruction with tendon graft allows for a large variety of graft fixation techniques. These can, according to Milano et al. be classified into compression, expansion and suspension types and in their prospective study all these different types of techniques were compared to each other in a clinical setting [11].

On the tibial side, Kousa et al. in an experimental study found superior strength in fixation of hamstring tendons with the Intrafix expanding bolt. Both biodegradable and metal interference screws failed at same values which were significantly lower compared to Intrafix. They also raised the question of whether bio and metallic interference screws provide adequate fixation strength in the post operative phase unless longer screws are used [12].

Aga et al. compared different screw and sheath devices with interference screws on the tibial side in a controlled experimental study. They found no significant differences between screws and sheat devices for ultimate failure load [13].

On the femoral side Kousa et al. found the Bone Mulch Screw to provide the strongest fixation followed by Endobutton and Rigidfix. The bioabsorbable Smart Screw provided an almost equal strength as the Rigifix, but the metallic interference screw failed at significantly lower values [14].

Persson et al. conducted a nationwide cohort study based on information obtained from the Norwegian Knee Ligament Registry, 2004–2013. In their study they compared the revision rate between patellar tendon (PT) and hamstring tendon (HT) grafts used in ACL reconstructions. They found a higher revision rate among reconstructions using the HT compared to PT graft reconstructions. HT fixations using extratunnel suspension technique (Endobutton) in combination with a biodegradable interference screw (Biosure HA) was found to have the highest revision rate. The lowest revision rate using the HT graft was seen with the combination of Intrafix and a metal interference screw. In our study on HT grafts were used and the suspension technique was used in two groups (group I and II) but the extracortical suspension technique was used in none of the groups. [15].

In a similar study earlier we were able to show that no statistical differences could be seen in the outcome values between fixation with cross-pin or metallic interference screw with hamstring tendons at a 5 years follow-up [16].

Eajazi et al. in their study compared three types of suspension fixation on the femoral side: Endobutton (cortical), Rigifix (corticocancellous) and Aperfix (cancellous). Their study included 96 patients randomly assigned to the study groups based on the order of referral to hospital. For clinical examination KT-1000 and Lysholm score were used and the follow-up time was 2 years. Aperfix yielded the best improvement in Lysholm score but no significant difference between the three groups was found [17].

Aydin and Ozcan compared three suspension types of fixation on the femoral side: Endobutton (cortical), TransFix (corticocancellous) and Aperfix (cancellous). They found that all three suspension types of fixation techniques led to significant improvements in knee performance with no differences between groups [18]. A prospective randomized study by Ibrahim et al. compared bioabsorbable Rigifix fixation with Endobutton fixation. They found a statistically significant difference in the instrumented knee laxity tests favoring cross-pin compared to Endobutton fixation (1.30 mm vs 1.95 mm p < 0.001). However no significant difference between the two groups was reported regarding the functional outcome [19].

There are multiple devices now available for fixing the hamstring graft and most clinical studies have failed to show any significant differences between these devices. However, even a reliable fixation device can perform badly if bone tunnels are malpositioned, technical failure related to the device has occurred or the post operative rehabilitation protocol is not strictly followed. In our study two experienced knee surgeons performed all the reconstructions and the rehabilitation program was identical in all four study groups. [20]

Our study has several limitations. There were altogether 4 violations to the study protocol. We did not want to exclude these cases, because these deviations were of minor importance and thought not to distort the principal results.

Although the patients and physiotherapists were blinded, the postoperative examinations were not performed by independent examiners and furthermore the examiners were not blinded to the fixation method used. Also, some of the 5-year follow-up results were obtained by mail, so all of the clinical parameters followed for this study were not recorded. There were 77 patients clinically evaluated at the 5-year follow-up (64,2%). Another limitation was that we concentrated only on clinical findings, radiological parameters such as tunnel position and tunnel widening were not included in this study.

The strength of our study is that this was a properly randomized prospective clinical study.

## Conclusions

In this study, 3 different techniques, compression, expansion and suspension fixation of the graft was used. Apart from additional procedures we could not detect any significant differences between the 4 groups at a minimum of 5 years of follow-up.

There was a statistically significant difference in additional procedures between the 2 and 5 years follow-up, group I RigidFix/IntraFix being the most likely method for an additional procedure.

All the fixation devices in this study are secure to use and provided improved patient performance.

## Data Availability

The data supporting the results reported in this article are kept and stored at the ORTON Orthopaedic Hospital, Helsinki, Finland.
